# Dosimetric impact of radiofrequency tumor treating field arrays on dose on surface and at depth

**DOI:** 10.1002/acm2.70704

**Published:** 2026-07-08

**Authors:** Sarah Ashmeg, James Taylor, Ron Lalonde, Shada Wadi‐Ramahi

**Affiliations:** ^1^ UPMC Hillman Cancer Center Pittsburgh Pennsylvania USA

**Keywords:** gliobastoma, optune, TTFields, tumor treating fields

## Abstract

**Background:**

Tumor Treating Fields (TTFields) have demonstrated a survival benefit in patients with glioblastoma and are increasingly being investigated for concurrent use with radiotherapy. However, published data regarding the dosimetric impact of TTFields transducer arrays during photon irradiation remain limited, particularly with respect to surface dose enhancement and attenuation at treatment depth. A clear, quantitative understanding of these perturbations is necessary to optimize clinical implementation.

**Purpose:**

This study aimed to directly quantify the physical dosimetric perturbations introduced by TTFields transducer arrays during megavoltage photon irradiation and to evaluate whether array repositioning mitigates surface dose enhancement and depth attenuation.

**Methods:**

An experimental measurement was conducted using a solid‐water slab phantom and a 6‐MV Varian TrueBeam linear accelerator with both flattened and flattening‐filter‐free (FFF) beams. An Optune 3 × 3 ceramic transducer array was placed on the phantom surface. Dose at depths of 1.5, 5, and 10 cm was measured using a Farmer‐type ionization chamber. Surface dose was assessed using silicon diodes, MOSFET detectors, and Gafchromic EBT3 film (referred to as radiochromic film in this paper). Measurements were performed with and without the array to establish baseline comparisons. To evaluate mitigation strategies, the array was shifted by one transducer diameter in four cardinal directions, and measurements were repeated. Dose ratios were calculated relative to baseline conditions without the array. Statistical significance was assessed where appropriate.

**Results:**

The presence of the TTFields array resulted in statistically significant attenuation at depth, with dose reductions of approximately 2%–4% up to 10 cm (*p* < 0.001). Surface dose increased substantially, with MOSFET and diode measurements demonstrating enhancement factors ranging from 1.81 to 1.87 relative to baseline. Radio film showed higher apparent enhancement, attributed to energy‐dependent over‐response in the high‐gradient surface region. Repositioning the array reduced peak surface dose by up to 40%. Film‐based dose profiles demonstrated a 24% reduction in localized hotspots following array shifts. Despite mitigation, residual surface dose remained elevated at approximately 1.6–2.3 times baseline values.

**Conclusions:**

TTFields transducer arrays introduce clinically meaningful dosimetric perturbations, characterized by significant surface dose enhancement, and measurable attenuation at depth. While array repositioning reduces localized hotspots, it does not eliminate elevated scalp dose.These findings underscore the importance of treatment‐specific dosimetric evaluation and careful surface dose assessment when delivering radiotherapy concurrently with TTFields.

## 1 INTRODUCTION

Glioblastoma multiforme (GBM), the most aggressive primary brain malignancy, accounts for approximately 54% of all gliomas with an incidence of 3.19 per 100 000 population.^1^ Despite decades of therapeutic advances, the prognosis remains dismal, with median overall survival of 14.6‐16.9 months following standard multimodal treatment.^2^ The current standard of care, established by the landmark EORTC‐NCIC CE.3 trial, consists of maximally safe surgical resection followed by concurrent chemoradiotherapy with temozolomide (60 Gy in 30 fractions with concurrent and adjuvant temozolomide).^3^ However, virtually all patients experience disease recurrence, necessitating the development and integration of novel therapeutic modalities.

**FIGURE 1:**
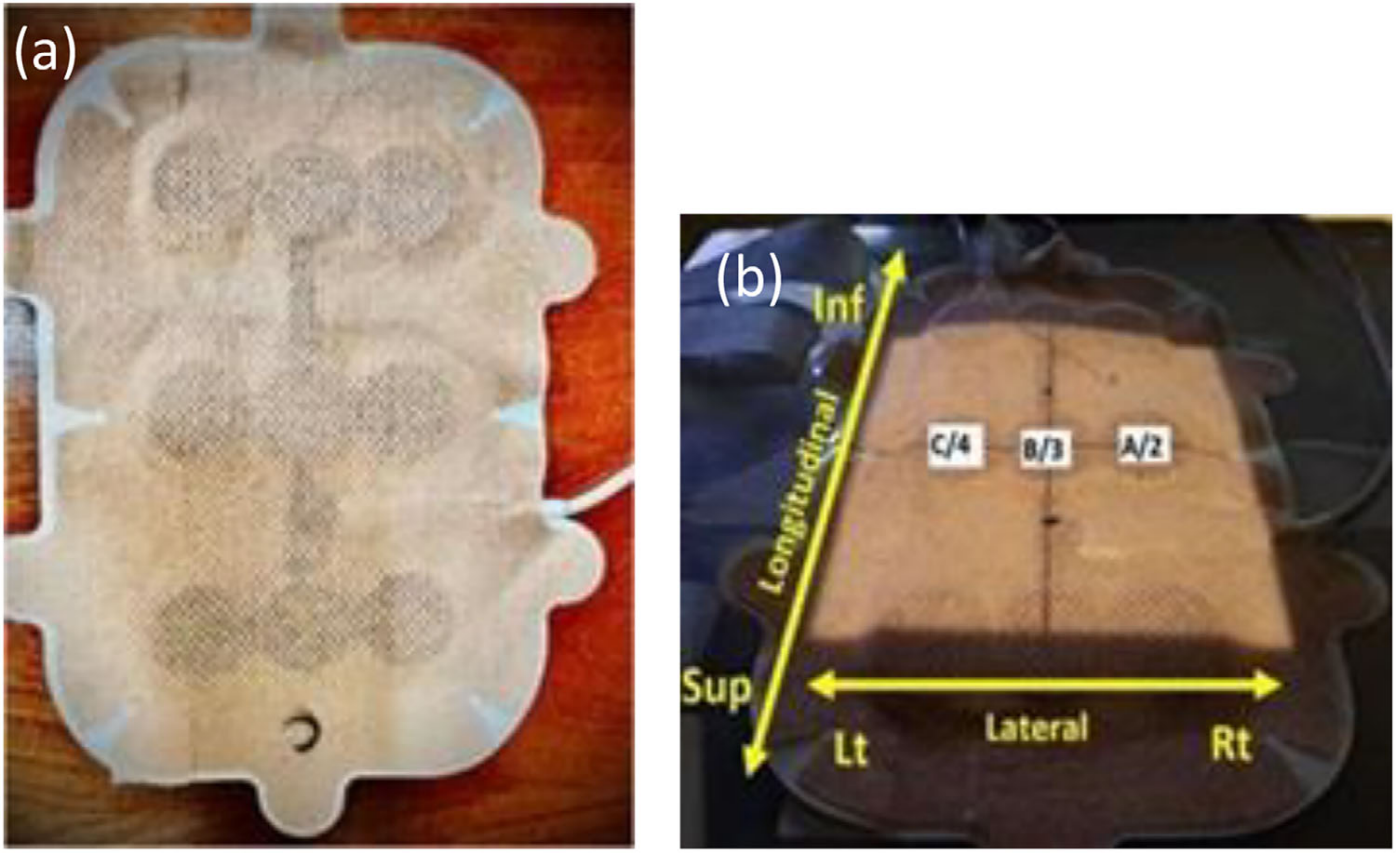
(a) Optune TTFields array used in this work. (b) Alignment of the Optune array under linac cross hair. The array was shifted by one electrode diameter (19 mm) in all 4 directions, the alphanumeric notations show the location of the diodes (alphabet) and MOSFETs (numbers) in the surface measurements.

Tumor Treating Fields (TTFields) represent a paradigm‐shift to cancer treatment, utilizing alternating electric fields at specific frequencies shown to disrupt cellular division through non‐thermal mechanisms.^4^ The technology employs low‐intensity (1‐3 V/cm), intermediate‐frequency (100‐300 kHz) alternating electric fields delivered via an array of ceramic transducer discs (Z ∼ 45–50) placed on the patient's skin. The Optune device (Novocure Ltd., Haifa, Israel), which delivers TTFields at 200 kHz for glioblastoma treatment, received FDA approval for treatment of recurrent GBM in 2011 and for newly diagnosed GBM in 2015 based on compelling clinical evidence.^5^ Figure 1(a) shows the clinical Optune array used in this work consisting of a 3 × 3 array of transducers.

A phase III clinical trial conducted in 2017 showed that the addition of TTFields resulted in statistically significant improvements in both progression‐free and overall survival compared to temozolomide alone. Importantly, the survival benefit was achieved without compromising quality of life, with the primary adverse event being mild to moderate skin irritation beneath the transducer arrays.^6^ More recent studies explored the potential synergistic effects of combining TTFields with radiotherapy, exploiting the enhanced cytotoxic effects observed with simultaneous application.^7^, ^8^, ^9^, ^10^ The studies showed that simultaneous applications are safe with only grade 1–2 skin toxicity. Skin toxicity is believed to be a combination of the adhesive nature of the patches in addition to the RF field and the enhanced radiation dose on the skin under the ceramic transducers. Localized high temperatures, as high as 41°C, have been reported leading to ulcers and infection requiring clinical care.^11^ To reduce the localized thermal spots, the manufacturer has advised patients to change the location of the transducer arrays twice a week.

The dosimetric studies on the effect of wearing the Optune device during radiation therapy treatment having been contradictory. One study^12^ used a cranial phantom with a circular lesion in the center of the phantom and transferred patients’ plans to the phantom to look at the effect of having the array on the plan DVH. The authors found an increased skin dose up to 3 %, with a build‐up in the first 10 mm similar to one layer of brass bolus, and insignificant changes in tumor's DVH. Another study came to a similar conclusion that the presence of the array resulted in clinically insignificant changes to the DVH of the CTV.^13^ On the other hand, Li et al^14^ used both measurement in a RANDO phantom as well as calculation of DVH to look at the dose at skin and at tumor depth in several VMAT plans. Their measurement found a localized dose increase to the skin by 70%–160%, and up to 2% dose reduction for the tumor at depth. The DVH from the plans showed similar dose reduction for the PTVs. Many factors could lead to contradictions in the reported results such as dose calculation algorithm used, dose grid resolution, whether density over‐rides were used, the plan complexity, and the DVH specifications.

All these factors make it difficult to isolate the effect of the transducer array. To understand the dosimetric effect of the Optune device, we studied the effects via a commissioning‐style setup and measured the effect of the device on the dose both localized and at depth using various measurement techniques and dosimeters. We also investigated how shifting the device affected surface dose and dose at depth.

To the best of our knowledge, this represents the first systematic, commissioning‐style dosimetric evaluation of the Optune transducer array; to date, no study has isolated the direct physical effect of the arrays using a detector‐agnostic approach while simultaneously quantifying both surface dose enhancement and attenuation at depth, including the impact of array positioning.

## 2 METHODS

TrueBeam linear accelerator (Varian Medical Systems, Palo Alto, CA) equipped with both conventional flattened and flattening filter‐free (FFF) photon beam capabilities was used for all measurements.

The TTFields device used in this study was an Optune transducer array consisting of a 3 × 3 configuration of ceramic discs. Each transducer disc has a diameter of 20 mm and thickness of 3 mm, containing high‐*Z* ceramic materials (primarily lead zirconate titanate, PZT). The array was positioned on the surface of a 22 cm solid water phantom with the central row of transducers aligned with the beam central axis at 100 cm SSD. The center of the middle electrode is positioned at the isocenter. Figure 1(b) shows the alignment of the Optune device with the linac's crosshair. A Farmer‐type thimble ionization chamber (PTW 30010, Freiburg, Germany) was used to measure dose at depth. Silicon diodes (Sun Nuclear, Melbourne, FL), MOSFETs (Best Medical Canada, Ottawa, Canada), and Radio‐chromic EBT3 film (Ashland Inc., Bridgewater, NJ) were used to measure surface dose. Effect of both flattened and FFF 6MV beams of 10 × 10 and 20 × 20 cm^2^ field sizes was measured. All detectors were calibrated according to institutional protocols and verified to be within expected tolerances; linac output was confirmed during routine morning QA and is known to be stable, minimizing the likelihood of output‐related variability in the measurements.

1. Depth dose measurement: The Farmer chamber was positioned in three depths of 1.5 cm (dmax for the energy used), 5 cm, and 10 cm within the solid water phantom. At each depth, a minimum of 10 cm backscattering was placed below the chamber's position.
2. Skin dose measurement: Surface dose measurements were performed using diodes, MOSFETs, and Radiochromic films. We opted to use multiple detectors for skin dose measurement to confirm that any measured differences are due to the presence of the Optune device.
3. Effect of array repositioning: The Optune manual (foot 1) requires the patients to move the Optune array twice a week to reduce skin‐related toxicities. To look at the effect on surface dose and at depth due to array relocation, the array was shifted by one transducer diameter (19 mm) in four cardinal directions: Superior, inferior, and both lateral directions, as shown in Figure 1(b). The film exposure protocol involved delivering 500 monitor units (MU) for the central position measurement and 100 MU to each of the five positions (central, superior, inferior, left, and right) for a total dose of 500 MU distributed across the positions.

Film calibration (H&D curve) was performed using known linac doses measured with a PTW TN30013 Farmer ionization chamber in solid water. The RED channel calibration curve spanned the dose range relevant to this study and extended beyond it. Calibration films were irradiated and scanned approximately 48 h post‐irradiation to allow for signal stabilization.

Film orientation was carefully controlled by marking a coordinate system on each film to prevent rotation or flipping during scanning and analysis. All films were handled and scanned under consistent conditions using an Epson Expression 10 000XL series flatbed scanner and RIT software (Radiological Imaging Technology, Inc., Colorado Springs, CO).

Measurements with diode, MOSFET, and film were performed sequentially under consistent setup conditions, with careful reproduction of detector positioning using fixed geometry and setup references.

Dose ratios were calculated as the ratio of dose measured with the TTFields array in place to the dose measured without the array (baseline condition). Statistical analysis included calculation ofmean values, standard deviations, and 95% confidence intervals for all measured parameters. In this setup, we are using different detectors to measure the dose in similar geometries. To test if the mean values calculated from each detector's response are similar, we performed a paired *t*‐test, with a significant *p*‐value set at 0.05.

## 3 RESULTS

1. Depth dose measurement

Central axis measurement using the Farmer chamber at different depths and for both flattened and FFF 6MV beams, showed that the average attenuation ratio over all depths was 0.962 ± 0.009 for 10 × 10 cm^2^ field size, Table 1(a) gives values for the measured ratios of dose with the Optune device to the dose without at each depth. A small but statistically significant (*p* < 0.001, paired *t*‐test) reduction in dose was observed at all depths, with no clinically meaningful depth dependence beyond buildup.

1. Surface dose measurement

**TABLE 1:** Dose ratios, measured as dose with the array to dose without it.

(a) At various depths. Farmer ion chamber was placed at isocenter for each depth with 10 cm backscatter material.			
Energy	1.5 cm	5 cm	10 cm
6MV	0.977	0.962	0.963
6FFF	0.963	0.952	0.955

(b) Average surface dose ratio in the presence of the array measured with multiple detectors (unshifted).	
Detector	Dose ratio
Diodes	1.87 ± 0.07
MOSFET	1.81 ± 0.11
Radiochromic films	2.89 ± 0.09

Surface dose was measured under the electrodes in positions A, B, and C in Figure 1(b). The average surface dose shows higher values in the presence of electrodes for all detectors used, Table 1(b). MOSFET measurements gave a dose ratio relative to open field of 1.81, similar to that of the diode's (*p* = 0.12). However, film measurement is 50% higher than the others, likely explained by films energy‐dependent over‐response to low‐energy electrons.^17^, ^18^

1. Effect of array repositioning

Shifting the array in the superior (positive‐value) and inferior (negative‐value) directions resulted in reduced surface dose, Table 2. Taking the average of all readings for both sup/inf shifts, the surface dose ratio is 1.16 ± 0.11. This represents a reduction of up to 40% from the non‐shifted dose in Table 1(b). At 10 cm depth, shifting of the array resulted in dose attenuation of < 0.99. The effect of the array depends on the field size, with larger field size resulting in lower overall values as compared to 10 × 10 cm2 field size (see Table 2) All results are statistically significant (*p* < 0.001).

**TABLE 2:** The dose for 10 × 10 cm^2^ field size: Measured with Farmer chamber & surface dose measured with diodes and MOSFETs.

Distance shifted (cm) sup‐inf	Chamber, *d* = 10 cm	Diode A	Diode B	Diode C	MOSFET 2	MOSFET 3	MOSFET 4
−1.9 (Inf)	0.9921	1.2937	1.2092	1.1142	1.3404	1.2167	1.0432
0 (No shift)	0.9622	1.7942	1.9221	1.8794	1.7594	1.9355	1.7440
1.9 (Sup)	0.9948	1.0986	1.1954	1.1049	1.0777	1.2258	1.0136

Distance shifted (cm) Rt ‐Lt	Chamber, *d* = 10 cm	Diode A	Diode B	Diode C	MOSFET 2	MOSFET 3	MOSFET 4
−1.9 (Left)	0.9659	1.1276	1.7805	1.8601	1.1146	1.7866	1.8640
0 (No shift)	0.9622	1.7942	1.9221	1.8794	1.7594	1.9355	1.7440
1.9 (Right)	0.9659	1.9314	1.8011	1.1439	1.8903	1.8280	1.0808

Table 3 shows how shifting the array left or right resulted in one diode becoming uncovered by the array. Taking the overall effect of the lateral shifts, the average surface dose ratio is 1.60 ± 0.36. The dose attenuation ratio at 10 cm depth has not changed from the unshifted configuration, staying at 0.966 differences that were not statistically significant (*p* > 0.05).

**TABLE 3:** The dose for 20 × 20 cm2 field size: measured with farmer chamber & surface dose measured with diodes and MOSFETs.

Distance shifted (cm) sup‐ inf	Chamber, *d* = 10 cm	Diode A	Diode B	Diode C
−1.9 (Inf)	0.9931	1.2233	1.1475	1.0872
0 (No shift)	0.9651	1.5178	1.6322	1.5810
1.9 (Sup)	0.9949	1.0756	1.1365	1.0803

Distance shifted (cm)Rt‐Lt	Chamber, *d* = 10 cm	Diode A	Diode B	Diode C
−1.9 (Left)	0.9682	1.1036	1.5020	1.5659
0 (No shift)	0.9651	1.5178	1.6322	1.5810
1.9 (Right)	0.9680	1.6416	1.5148	1.1115

**FIGURE 2:**
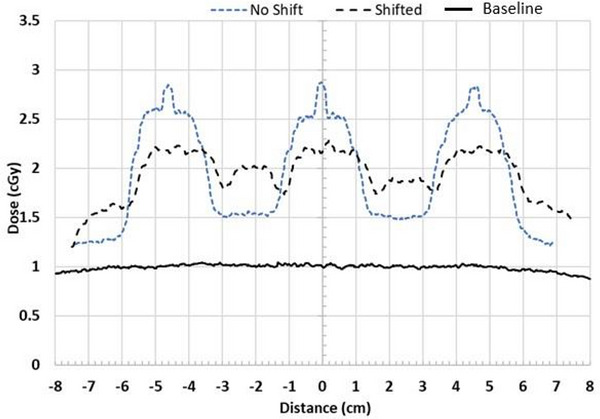
Profiles for a 10 × 10 cm^2^ field size taken with films placed on top of the phantom. The black solid profile plots film dose for an open field without the array placed, this is the baseline. The blue dashed profile plots dose with the array placed stationary on top of the film (6MV), and the black dashed lines represent the profile of the Optune devices shifted in all four directions.

Film analysis provides a two‐dimensional visualization of the dose distribution beneath the transducer discs and in the gaps between them. Figure 2 shows dose profiles derived from three film conditions: (1) baseline (open field, no array), (2) unshifted array, and (3) shifted array.

For the unshifted array film, the same monitor units were delivered as in the baseline (open field) film. Relative to baseline, the dose beneath each transducer disc was increased by a factor of 2.5–2.6. Additionally, a localized peak was observed at the center of each transducer disc, reaching approximately 2.9 times the baseline dose. These central spikes are likely attributable to structural components within the center of each disc.

The regions between adjacent transducers exhibited a dose increase of approximately 1.5 times the baseline. This enhancement is likely due to the presence of connecting wires within the array, which are oriented along the direction where the increase was observed, in addition to a minor contribution from scattered radiation.

The shifted array profile (black dashed line in Figure 2) represents a composite irradiation designed to simulate clinical repositioning of the arrays. In this configuration, the film remained stationary while irradiation was delivered in five geometries: one unshifted position and four shifted positions in the cardinal directions. A total of 500 monitor units were delivered (100 MU per position). Compared to the unshifted configuration, array shifting resulted in a spatial blurring of the dose distribution, with peak dose reduced by 24% (*p* < 0.001). Despite this reduction, the dose across the irradiated area remained elevated at approximately 1.6–2.3 times higher than the baseline (open field) condition.

**FIGURE 3:**
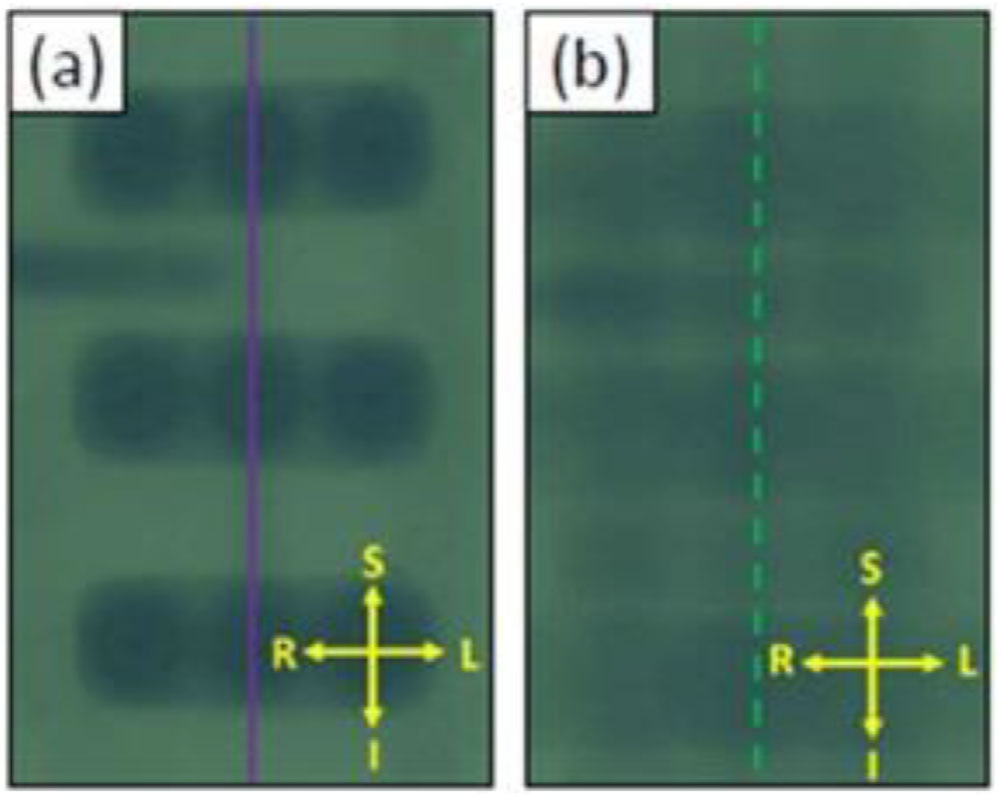
2D films scans taken with (a) Array stationery, and (b) Array centered then shifted in 4 = four cardinal directions. The line in the middle of each film approximates the location of the profiles in Figure 3.

Figure 3 shows the two films used. Figure 3 (a) is the film irradiated with the array in place, while Figure 3 (b), shows the film after irradiating it with the array in five total locations. The circular high dose areas under each transducer were less pronounced with the shifts. However, shifting the transducers meant that other areas also received localized high doses, and the overall effect is an increase in dose in the entire field by 1.6‐2.3 times compared to dose without the array.

## 4 DISCUSSION

Previous investigations into the dosimetric impact of TTFields arrays have reported inconsistent findings regarding surface dose and attenuation at depth. Two studies^12^, ^13^ concluded that the presence of TTFields arrays resulted in minimal perturbation of target volume coverage and only modest surface dose increases. In contrast, another study^14^ reported highly localized surface dose enhancements ranging from 70%–160% and up to 2% dose reductions at tumor depth using both RANDO phantom measurements and DVH analyzes. These discrepancies have been attributed to variations in phantom geometry, dose calculation algorithms, and handling of material heterogeneities, but no study to date has isolated the direct physical effect of the transducer arrays using a commissioning‐style, detector‐agnostic approach.

In this work, we addressed these limitations by employing a controlled slab‐phantom geometry and multiple, independent dosimetry systems to directly quantify the physical perturbation introduced by the array. At depths up to 10 cm, ion chamber measurements showed a statistically significant attenuation of approximately 2%–4% for both flattened and FFF beams (*p* < 0.001, paired *t*‐test), as shown in the Results section.

At the surface, measurements obtained using MOSFETs, diodes, and radiochromic film revealed detector‐dependent differences: MOSFETs and diodes showed a mean dose enhancement of approximately 1.8, whereas radiochromic film demonstrated substantially higher apparent enhancement (approximately 50% higher), likely due to its over‐response to low‐energy electrons. In addition, detector construction contributes to these differences through their effective measurement depth within the buildup region: the Sun Nuclear skin (black) diodes have an inherent buildup of approximately 1 mm, whereas radiochromic film has a thickness of approximately 0.275 mm. As a result, radiochromic film measurements more closely represent true surface dose, while diode measurements reflect dose slightly deeper along the percent depth dose (PDD) curve, contributing to the observed differences. No radiochromic film measurements were performed at depth. Although measurements were not performed simultaneously, potential positioning uncertainties are expected to be minimal and unlikely to impact the observed dosimetric trends, as all detectors were used to support relative comparisons. In addition, while film calibration and handling followed established protocols, additional characterization of absolute dose response and extended validation of film dosimetry were beyond the scope of this study and represent areas for future investigation.

This multi‐detector agreement supports the presence of dose perturbations introduced by the high‐*Z* ceramic transducers, consistent with increased electron fluence and localized buildup effects, although the specific contribution of scatter cannot be independently confirmed with the current measurement setup. Importantly, our systematic evaluation of array repositioning provides new quantitative insight into a manufacturer‐recommended mitigation strategy that has not previously been studied dosimetrically. Shifting the array one transducer diameter in four cardinal directions reduced peak surface dose enhancements by up to 40%, with film profiles showing a 24% reduction in localized hotspots. However, even after repositioning, residual surface doses remained 1.6–2.3 times higher than baseline, indicating that repositioning alone does not eliminate the risk of significant scalp dose escalation. These findings provide the first experimental evidence that while array shifting may reduce peak toxicity‐driving hotspots, it also redistributes excess dose over a broader scalp region, a phenomenon not previously described in the literature.

Clinically, these findings shift the paradigm from hotspot‐centric toxicity mitigation to a more comprehensive consideration of dose redistribution across the scalp. While array shifting may reduce peak dose regions, the associated spread of dose over a larger surface area may increase the risk of diffuse dermatitis, necessitating a balance between peak dose reduction and exposed area. These results support the need for patient‐specific array positioning strategies that account for beam geometry and individual anatomy. However, as this study was conducted using simplified beam geometries, translation to clinical practice, where patients are predominantly treated with VMAT, requires careful consideration, and the dosimetric impact of array shifting should be evaluated within modulated treatment deliveries. Collectively, this underscores the importance of treatment‐specific dosimetric assessment and may warrant reconsideration of scalp dose metrics beyond maximum dose when Optune is used concurrently with radiotherapy.

## 5 CONCLUSIONS

Using a commissioning‐style experimental setup, we quantified the dosimetric impact of the Optune transducer array, demonstrating 2%–4% attenuation at depths up to 10 cm and surface dose enhancement of approximately 1.8× as measured by MOSFETs and diodes. Array repositioning reduced localized high‐dose regions; however, residual surface dose remained elevated (approximately 1.6–2.3× baseline).

Given that dermatologic toxicities are among the most commonly reported adverse effects in patients receiving TTFields concurrently with radiotherapy,^15^, ^16^ understanding the impact of TTFields arrays on surface dose remains clinically relevant. Our findings demonstrate that while array repositioning may reduce localized hotspots, it does not eliminate elevated surface dose and may instead redistribute dose over a broader surface area.

Based on these findings, we recommend that clinical teams: (1) evaluate the feasibility of treatment delivery without the array when appropriate, (2) coordinate array repositioning to promote dose averaging across treatment fractions, (3) perform treatment‐specific measurements to assess both surface dose and depth attenuation, and (4) evaluate the ability of clinical dose calculation methods to reproduce these effects to support patient‐specific decision‐making.

## AUTHOR CONTRIBUTIONS

**Sarah Ashmeg**: Conceptualization; study design; data curation; formal analysis; development of figures and visualizations; writing of original draft; manuscript review & editing. **James Taylor**: Study design; data collection; validation; development of figures and visualizations; manuscript review & editing. **Ron Lalonde**: Clinical expertise; resources; manuscript review & editing. **Shada Wadi‐Ramahi**: Conceptualization; guidance on study design; formal analysis; critical review of data interpretation; substantial editing and rewriting of the manuscript; supervision.

## CONFLICT OF INTEREST STATEMENT

The authors declare no conflict of interest.
